# The Social and Natural Environment’s Impact on SARS-CoV-2 Infections in the UK Biobank

**DOI:** 10.3390/ijerph19010533

**Published:** 2022-01-04

**Authors:** Ryan J. Scalsky, Yi-Ju Chen, Zhekang Ying, James A. Perry, Charles C. Hong

**Affiliations:** 1 Medical Scientist Training Program, University of Maryland School of Medicine, Baltimore, MD 21201, USA; ryan.scalsky@som.umaryland.edu; 2 Department of Medicine, University of Maryland School of Medicine, Baltimore, MD 21201, USA; Yvette.Chen@som.umaryland.edu (Y.-J.C.); zying@som.umaryland.edu (Z.Y.)

**Keywords:** socioeconomic status, air pollution, SARS-CoV-2, COVID, UK biobank

## Abstract

COVID-19 has caused a global pandemic with considerable impact. Studies have examined the influence of socioeconomic status and air pollution on COVID-19 risk but in low detail. This study seeks to further elucidate the nuances of socioeconomic status, as defined by the Index of Multiple Deprivation (IMD), air pollution, and their relationship. We examined the effect of IMD and air pollution on the likelihood of testing positive for SARS-CoV-2 among 66,732 UKB participants tested for SARS-CoV-2 from 16 March 2020 through 16 March 2021. Logistic regression was performed controlling for age, sex, ancestry and IMD or air pollution in the respective models. IMD and its sub-scores were significantly associated with increased risk of testing positive for SARS-CoV-2. All particulate matter less than 2.5 μm (PM2.5), nitrogen oxide (NO_x_), and nitrogen dioxide (NO_2_) levels were associated with increased likelihood of testing positive for SARS-CoV-2. Measures of green space and natural environment around participants’ homes were associated with reduced likelihood of SARS-CoV-2. Socioeconomic status and air pollution have independent effects on the risk of testing positive for SARS-CoV-2. Green space and natural environment space in the proximity of people’s homes may mediate the effect of air pollution on the risk of testing positive for SARS-CoV-2.

## 1. Introduction

In December 2019, a novel coronavirus began rapidly spreading throughout eastern central China focused around the city of Wuhan [1]. This virus, severe acute respiratory syndrome coronavirus 2 (SARS-CoV-2), has since developed into a global pandemic now actively infecting 71.5 million people [2]. With few signs of slowing down, the need to identify risk factors that predispose individuals to contracting SARS-CoV-2 becomes increasingly vital. Preliminary studies have examined biological and behavioral factors that may contribute to SARS-CoV-2 risk, such as smoking, lipid levels, BMI, and pre-existing conditions [3,4,5] but little attention has been paid to environmental and social variables. Though socioeconomic status and air pollution have both been demonstrated to play critical roles in a variety of disease pathogenesis, we know nearly nothing of their impact on SARS-CoV-2 susceptibility [6,7]. Preliminary studies have demonstrated that areas of low socioeconomic status (SES) have increased positive testing rates and worsening outcomes than those of higher SES [8]. Additionally, metrics of air pollution have been associated with increased fatality in numerous countries [9]. These results indicate a need for further exploration of both the effect of SES and air pollution but also how their interaction may function to increase SARS-CoV-2 susceptibility. In this paper, we further characterize the impact of socioeconomic status, air pollution and their relationship on SARS-CoV-2 susceptibility. 

## 2. Materials and Methods

The UK Biobank is a large, ongoing prospective cohort study that recruited over 500,000 UK participants between 2006–2010, ranging in age from 40–69 years at the time of recruitment. Extensive health-related records were collected from these participants, including socioeconomic status scores and environmental measurements. In April 2020, the UK Biobank resource began releasing SARS-CoV-2 test results to approved researchers. This study leveraged available data based on test results taken during the 12-month period of 16 March 2020 through 16 March 2021. Participants consented to the use of their data through UK Biobank protocols. The data were fully de-identified prior to distribution to approved researchers. Full details on these test results are available online [10]. 

Participants testing positive for SARS-CoV-2 were considered cases. If a participant had more than one test, we classified that participant as a case if any test gave a positive result. This was done because false positives are less likely than false negatives, with regard to these tests. Participants who only had negative test results were classified as controls. Though the initial testing was restricted to hospital settings in symptomatic individuals, from 27 April 2020 and onward, testing was expanded to include community clinics and all non-elective patients admitted to facilities regardless of symptoms. Almost all tests discussed in this study were SARS-CoV-2 RNA based PCR tests, collected through nose and throat swabbing. 

Analyses were performed using Plink2, logistic regression [11]. “SARS-CoV-2 test status” was a binary variable which was run against continuous variables, and their sub-scores, representing socioeconomic status and environmental variables for air quality, greenspace and natural environment space. All data were supplied by the UK Biobank and is based on participant’s home location at the time of enrollment in the UK Biobank (2006–2010). Covariates of sex, age and principal components (PCs) 1 through 5, to adjust for ancestry, were included in all models. Principal component analysis is a standard technique used in statistical genetics which generates a dataset of PCs that can be used as covariates to correct for population stratification (i.e., differences in ancestry) [12]. The PCs used in this study were provided by the UK Biobank, which were computed from the cohort’s genotypes. Preliminary analysis showed that the first 5 PCs were significant at *p* < 0.05 and thus were included as covariates. Additional covariates were included in certain analysis models. Our analysis of air pollution and environmental measures included the Index of Multiple Deprivation (IMD) as a covariate to adjust for socioeconomic status. Our analysis of socioeconomic scores and environmental measures included both particulate matter less than 2.5 μm (PM2.5) and nitrogen oxide (NO_x_) levels as covariates to adjust for air pollution.

Odds ratios (OR) and 95% confidence intervals (CI) were generated for each trait tested against the “SARS-CoV-2 test status”. The continuous phenotypes were scaled to adjust the standard deviation to a value of 1.0, and thus the OR indicates the odds per standard deviation increase in the continuous phenotype. Given that one represents no increased or decreased odds, ORs greater than one indicated higher odds of SARS-CoV-2 positive test and ORs less than one indicate a lower odds of testing positive for SARS-CoV-2.

Air pollution estimates from 2005 to 2007 where derived from EU-wide air pollution maps with a resolution of 100 m × 100 m. Maps were then modeled using land use regression models which included satellite derived air pollution estimates (variables included: NO_2_ 2005, 2006 and 2007 and PM10 2007). Air pollution estimates for 2010 were modeled using land use regression developed in conjunction with the European Study of Cohorts of Air Pollution Effect (ESCAPE). Values were based on ESCAPE’s physical monitoring done in 37 locations between January 2010 and January 2011 combined with land use regression (variables included: NO_2_ 2010, NO_x_ 2010, PM10 2010 and PM2.5 2010). More information available on ESCAPE monitoring (https://cordis.europa.eu/docs/results/211/211250/117238471-6_en.pdf; accessed on 23 December 2021) and more information on UK Biobank air pollution variables (https://biobank.ctsu.ox.ac.uk/crystal/ukb/docs/EnviroExposEst.pdf; accessed on 23 December 2021). 

Index of Multiple Deprivation and its respective sub-scores were defined by census data collected by the UK Government overlayed onto geo-spatial data that defined the areas examined (https://assets.publishing.service.gov.uk/government/uploads/system/uploads/attachment_data/file/833947/IoD2019_Research_Report.pdf; accessed on 23 December 2021).

## 3. Results

### 3.1. Demographics 

This dataset includes 15,156 cases and 51,576 controls for a total of 66,732 subjects. Prior to the association analysis we compared the cases and controls for differences in sex, ancestry, and age. Significant differences were found between the ancestry (*p*-value = 2.8 × 10^−100^; Table 1) and age (*p*-value < 1 × 1010^−300^; Table 1) of cases and controls.

### 3.2. Socioeconomic Status 

This study found that lower socioeconomic status was associated with increased risk of contracting SARS-CoV-2. Index of Multiple Deprivation (IMD) was associated with an increase likelihood of testing positive for SARS-CoV-2 (OR = 1.207, 95% CI = 1.18–1.23, *p*-value = 1.4 × 10^−77^; Table 2). All sub-scores of IMD were associated with increased risk of SARS-CoV-2 testing risk (Table 2). Health score (OR = 1.261, 95% CI = 1.24–1.29, *p*-value = 6.8 × 10^−103^; Table 2), Employment score (OR = 1.207, 95% CI = 1.19–1.23, *p*-value = 3.1 × 10^−83^; Table 2), Education score (OR = 1.198, 95% CI = 1.18–1.22, *p*-value = 8.4 × 10^−82^; Table 2), Income score (OR = 1.179, 95% CI = 1.16–1.20, *p*-value = 5.9 × 10^−60^; Table 2), Living environment score (OR = 1.106, 95% CI = 1.08–1.13, *p*-value = 5.1 × 10^−21^; Table 2), Crime score (OR = 1.102, 95% CI = 1.08–1.12, *p*-value = 2.6 × 10^−20^; Table 2), Housing score (OR = 1.066, 95% CI = 1.05–1.09, *p*-value = 5.9 × 10^−10^; Table 2).

### 3.3. Air Pollution 

Prior studies have demonstrated that a wide variety of air pollution metrics including nitrogen dioxide, nitrogen oxides and particulate matter are associated with increases in SARS-CoV-2 risk and severity of infection [13]. While controlling for age, sex, 5pc and IMD our data reinforces these findings and elucidates nuances not previously reported. Less than 2.5 μm particulate matter (PM2.5) levels recorded in 2010 were significantly associated with an increase SARS-CoV-2 positive testing likelihood (OR = 1.063, 95% CI = 1.04–1.09, *p*-value = 5.58 × 10^−9^; Table 3). Nitrogen oxide (NO_x_) levels from 2010, which include nitrogen dioxide, were significantly associated with increase SARS-CoV-2 infection likelihood (OR = 1.067, 95% CI = 1.05–1.09, *p*-value = 1.66 × 10^−10^; Table 3). Nitrogen dioxide (NO_2_) levels recorded in 2005 (OR = 1.048, 95% CI = 1.02–1.07, *p*-value = 8.95 × 10^−6^; Table 3), 2006 (OR = 1.068, 95% CI = 1.04–1.09, *p*-value = 5.23 × 10^−10^; Table 3), 2007 (OR = 1.035, 95% CI = 1.01–1.06, *p*-value = 0.001; Table 3) and 2010 (OR = 1.081, 95% CI = 1.06–1.10, *p*-value = 3.43 × 10^−13^; Table 3) were all associated with an increase in SARS-CoV-2 positive testing likelihood. 

### 3.4. Green Space 

When controlling for IMD, green space measured 300 m (OR = 0.925, 95% CI = 0.906–0.944, *p*-value = 1.99 × 10^−13^; Table 4) and 1000 m (OR = 0.940, 95% CI = 0.920–0.960, *p*-value = 7.85 × 10^−9^; Table 4) around participants homes significantly reduced the chance of testing positive. This effect was smaller, but remained significant at 300 m (OR = 0.961, 95% CI = 0.937–0.985, *p*-value = 0.002; Table 4) and 1000 m (OR = 0.957, 95% CI = 0.933–0.981, *p*-value = 6.29 × 10^−4^; Table 4) when controlling for air pollution (PM2.5 and NOx) instead of IMD. Additionally, the trend persisted with natural environment at 300 m (OR = 0.939 95% CI = 0.920–0.960 *p*-value = 3.57 × 10^−9^; Table 4) and 1000 m (OR = 0.941, 95% CI = 0.921–0.961, *p*-value = 2.70 × 10^−8^; Table 4) around participants homes when controlling for IMD. This effect also diminished but remained significant when controlling for air pollution instead of IMD at 300 m (OR = 0.974, 95% CI = 0.950–0.999, *p*-value = 0.04; Table 4) and 1000 m (OR = 0.952, 95% CI = 0.928–0.977, *p*-value = 1.76 × 10^−4^; Table 4)

## 4. Discussion

Multiple studies have now examined the impact of socioeconomic status, usually defined as household income and education level, on the risk of contracting SARS-CoV-2 [14,15,16]. These simplified measures of socioeconomic status are wieldy, but of low resolution and do not accurately capture the multifaceted nature of socioeconomic status. We found that all sub-scores of the IMD significantly affected the likelihood of testing positive for SARS-CoV-2 but not with the same contributions. To little surprise, the most relevant sub-score of the IMD was the health score (OR = 1.261), which, as a composite measure of illness, disability, and medical comorbidities, aligns with prior research suggesting that comorbidities, especially obesity and cardiovascular disease increase risk of COVID-19 [6]. Interestingly, education and employment scores had the next highest odds ratios (OR = 1.198 and OR = 1.207, respectively). The education score is a measure of deprivation in education, skills, and training relating to one’s ability to work. It is suspected that these lower education scores correlate to jobs that do not provide benefits like working from home or paid leave, and have an older working population, as is the case with many minimum wage jobs [17,18,19,20,21]. Prior research has demonstrated these minimally educated workers are more likely to be exposed to SARS-CoV-2 with their low wages demanding they work despite the risk [19,20,21]. The employment score defines deprivation by the degree of involuntary exclusion from the labor market such as in cases of disability or unfortunate life circumstances. It is possible that this sub-score captures those who are homeless or unable to acquire testing, or basic medical care. It has been established that the non-employed and homeless communities are at heightened risk of contracting SARS-CoV-2 [22,23]. The income, housing and living environment scores likely capture similar trends. As individuals are less able to socially distance, maintain income, and get access to testing and basic health care, they are more likely to be exposed to SARS-CoV-2 regardless of their intents [20,21,22]. These results demonstrate that the constituent factors of socioeconomic status do not contribute equally to its effect on SARS-CoV-2 risk. Furthermore, they highlight the need to examine socioeconomic status and its contributing factors with higher resolution. The issue of mitigating deleterious effects of socioeconomic status on health is as old as modern research, and though great strides have been made, serious shortcomings exist. We believe that by targeting the most significant contributing factors, public health decisions can be made that more effectively mitigate poor socioeconomic status’ deleterious effects on health. 

Air pollution is another factor that affects the population as ubiquitously as socioeconomic status. In the context of respiratory illness and even specifically SARS-CoV-2, the effects of air pollution have been well defined [9]. Our study reiterates the increased risk of SARS-CoV-2 associated with high air pollution, but also sought to understand the interaction air quality may have with socioeconomic status. Exactly how impactful PM2.5 is on SARS-CoV-2 risk is not entirely clear, though studies have demonstrated that PM2.5 has unique properties that differentiate it from other air pollution metrics [13,24,25]. PM2.5 has been found to be able to cross the alveolar membranes and enter the blood, which allows it to exert effects beyond the mechanical [25,26]. Studies have demonstrated that PM2.5 can modulate the immune system and predispose individuals to or exacerbate respiratory illness [26,27]. It may be that this immune modulation, in addition to the mechanical stress that particulate matter exert, predisposes individuals to SARS-CoV-2. Lastly, higher measurements of NO_x_ and NO_2_ were found to increase the likelihood of an individual testing positive for SARS-CoV-2. Prior research has shown exposure to NO_2_ and NO_x_ can increase mortality associated with cardiovascular and respiratory conditions, as well as worsened outcomes in viral infections [28,29,30]. Furthermore, studies have examined the influence of air pollution on SARS-CoV-2 pathogenesis, finding that exposure may reduce mucociliary clearance, epithelial permeability, and immune cell function [31]. Our findings corroborate those from previous studies, and we believe these effects may predispose individuals to SARS-CoV-2 infection. 

The World Health Organization (WHO) sets Air Quality Guidelines (AQG) based on previous evidence of mortality associated with short and long-term exposure to air pollutants. The WHO AQG sets recommendations for which they claim defines cut-off points that effectively negate any attribution of pollution to mortality. The WHO AQG recommendation for PM2.5 is 5 micrograms/m^3^ per year and their reports claim a relative risk of 1.08 for every 10 microgram/m^3^ increase in PM2.5 [32]. This indicates that in this population PM2.5 levels are twice that of the AQG recommendation and likely contribute to mortality and potentially susceptibility to disease. The AQG for PM10 is 15 micrograms/m^3^ per year [32]. Though our analyses only found PM10 to be significantly associated with testing positive for SARS-CoV-2 in 2010, in both years measured, PM10 values exceeded the WHO AQG recommendation. This suggests that a higher resolution analyses of PM10’s contributions to SARS-CoV-2 risk is warranted. Lastly, WHO AQG guideline for nitrogen dioxides is 10 micrograms/m^3^. Though our results show a downward trend in NO_2_ levels, all years examined exceeded the AQG recommendations. This provides further support that reported levels of NO^2^ may increase SARS-CoV-2 susceptibility or mortality [32]. 

In each model, socioeconomic status and air pollution, the opposite was controlled for. Despite this, all sub-scores of the IMD and NO_2_ and NO_x_ recorded in 2010 remained significant. This indicates that though there is interaction between IMD and air pollution variables and the effects of both stand significant and independent of each other. The effect of IMD, as compared to air pollution variables, on green space in reducing SARS-CoV-2 positive testing likelihood is not equivalent, as indicated by differences in odds ratios and prior modeling. Despite this, our analyses demonstrated significance in all models. This suggests that green and natural environment space might mitigate some of the effect of air pollution on odds of testing positive for SARS-CoV-2 but further, more targeted analyses would be required to better understand these relations. 

These results demonstrate that socioeconomic status and air pollution both have significant and independent effects on likelihood of testing positive for SARS-CoV-2. Additionally, this data suggests that green space may mitigate some of the effect of air pollution on SARS-CoV-2 positivity rates. These results indicate that defining socioeconomic status more precisely may be fruitful in mitigating its deleterious effects on COVID-19 disease development. Lastly, the use of green space in cities may be effectively offsetting the impact of air pollution on proximal populations. These populations tend to be those of lower socioeconomic status [33,34] and so a dual pronged approach of implementing public health policy aimed at specific aspects of socioeconomic status with urban planning that incorporates selectively placed green space may provide a more efficacious approach to mitigating and ultimately improving the health of low socioeconomic status residents, especially with regard to risk for respiratory illness. 

Though these results are informative, it is essential to address limitations in the study design and understand how they impact our interpretation of the results. Association studies are always subject to sampling bias. Most importantly, this study lacks homogeneity in the type of SARS-CoV-2 test used and the severity of each case. We do not believe variability in testing type to be a major confound as the vast majority of tests were performed with PCR on samples from nose/throat swabs. Additionally, we recognize that the outcome in this study is a positive test which is not definitive proof of infection. Given that the majority of tests were PCR tests, which have high sensitivity and specificity if performed correctly, we believe this to be a sufficient stand-in for SARS-CoV-2 infection [35]. Another limitation is that the most recent data for air pollution was collected in 2010. A consistent temporal effect was noted but lack of more recent data prevents any comprehensive understanding of the potential longitudinal effects of air pollution of SARS-CoV-2 infection risk. This likely does not impact the findings as previous studies have identified that air pollution exposure can result in sequelae years or even decades later, suggesting that not all effects are resolved with time, but the nature of exactly how long that time frame is in the context of SARS-CoV-2 is unknown [36,37,38]. Not only this but the UK government has tracked air pollution quality in England from 1992 to 2020. They’ve found that PM2.5 were approximately 7.88 micrograms/m^3^ in 2020 in urban background environments and 8.06 micrograms/m^3^ at roadside. They found PM10 levels to be 13.22 micrograms/m^3^ in 2020 in urban background environments and 16.33 micrograms/m^3^ in urban traffic. Lastly, they found nitrogen dioxide levels to be 15.06 micrograms/m^3^ in 2020 in urban background environments and 22.95 micrograms/m^3^ at roadside [39,40]. All of these values exceed the WHO AQG recommendations in at least one location, which are based on levels that increase risk of mortality associated with excess air pollution [32]. Additionally, worth considering is that these levels likely do not indicate the most accurate depiction of air pollution levels that individuals were subject to prior to the start of the pandemic. Multiple studies have shown that air pollution has significantly reduced as a result of pandemic measures and so 2020 measurements likely under-estimate air pollution in England [41,42]. Given that recent estimates still exceed WHO AQG recommendations, we believe the results found in our analyses remain relevant and valid. Finally, we acknowledge that the IMD scores and sub-scores were collected between 2006 and 2010. Although these measurements were collected at least 10 years prior to the pandemic, we believe their fundamental relation to SARS-CoV-2 susceptibility is legitimate for the following reasons. Firstly, socioeconomic status has been shown to have robust, long-term effects on health. In particular, low SES is associated with increased prevalence of obesity, hypertension, diabetes mellitus and smoking, all of which are known to increase susceptibility to SARS-CoV-2 [43,44,45,46]. Furthermore, poverty rates have remained stable in the UK over the last ten years, indicating relatively stagnant social mobility [47,48]. Taken together, these trends suggest that our findings are likely still relevant within the sample and bolster the study’s generalizability. 

## 5. Conclusions 

In conclusion, we believe that despite these limitations this study provides a unique perspective on the driving components of socioeconomic status on disease development. Additionally, it clarifies the relationship between socioeconomic status and air pollution and provides ways that urban planning may provide health benefit to proximal populations. We suggest that future studies further define the relationship between socioeconomic status and air pollution, especially in how they interact, and how this interaction impacts disease development. Though the relationship between green space and air pollution seems promising, future studies should seek to validate these conclusions and identify community data that may shed light on the impact of urban planning decisions in negating the effects of air pollution on an area’s population. Such urban planning and mitigation efforts might be impactful given the likelihood that COVID-19 will become endemic in the future. 

## Figures and Tables

**Table 1:** Demographics—Sex, Ancestry and Age.

	N	Male (%)	Female (%)	White (%)	Non-White (%)	Age (SD)
Cases	15,156	7200 (47.5)	7956 (52.5)	13,545 (89.4)	1611 (10.6)	65.32 (8.61)
Controls	51,576	24,094 (46.7)	27,482 (53.3)	48,645 (94.3)	2931 (5.7)	69.64 (7.97)
All	66,732	31,294 (46.9)	35,438 (53.1)	62,190 (93.2)	4542 (6.8)	68.66 (8.32)
% positive	22.7%	23.0%	22.5%	21.8%	35.5%	N/A
*p*-value		8.6 × 10^−2^		2.8 × 10^−100^		<1 × 10^−300^

N/Male/Female/Non-white/White indicate number of subjects. Age is the mean age as of 2020, SD is standard deviation. *p*-values are from chi-squared test for sex and ancestry, and *t*-test for age, comparing cases and controls. White ancestry includes subjects self-reporting as White, British, Irish, or “Any other white background”. Non-white ancestry includes all other self-report categories.

**Table 2:** Effect of Socioeconomic Status.

Measure ^1^	Cases	Controls	Range ^2^	Mean (SD)	Odds Ratio ^3^	*p*-Value	95% CI
Health Score	13,957	49,173	−3.1–3.79	−0.070 (0.883)	1.261	6.8 × 10^−103^	1.24–1.29
Employment Score	13,957	49,173	0–0.75	0.091 (0.062)	1.207	3.1 × 10^−83^	1.19–1.23
Education Score	13,957	49,173	0.02–98.09	16.37 (16.62)	1.198	8.4 × 10^−82^	1.18–1.22
Income Score	13,957	49,173	0.01–0.77	0.120 (0.101)	1.179	5.9 × 10^−60^	1.16–1.2
Living Environment Score	13,957	49,173	0.08–92.99	18.63 (15.35)	1.106	5.1 × 10^−21^	1.083–1.130
Crime Score	13,957	49,173	−2.73–3.81	−0.027 (0.782)	1.102	2.6 × 10^−20^	1.08–1.12
Housing Score	13,957	49,173	0.34–70.14	19.64 (10.20)	1.066	5.9 × 10^−10^	1.05–1.09
Index of Multiple Deprivation (IMD)	13,957	49,173	0.61–82	18.24 (14.35)	1.207	1.4 × 10^−77^	1.18–1.23

Covariates: Age, Sex, 5 principal components, NOx, PM2.5. ^1^ Scores indicate deprivation level with higher values indicating more deprivation. Housing scores from UK Biobank were adjusted (inverted) to be consistent with this definition. ^2^ Range of variables throughout entire UK Biobank data. ^3^ Odds Ratio of testing positive for COVID19 for a one standard deviation increase in deprivation score.

**Table 3:** Effect of Air Pollution.

Measure	Cases	Controls	Mean (SD)	Odds Ratio	*p*-Value	95% CI
PM 2.5–2010	13,957	49,173	10.0 (1.05)	1.063	5.58 × 10^−9^	1.04–1.09
PM 10–2010	13,957	49,174	16.25 (1.90)	1.014	0.011	1.01–1.02
PM 10–2007	13,940	49,097	22.47 (2.80)	0.9965	0.346	0.99–1.01
NO_x_–2010	13,957	49,185	44.13 (15.81)	1.067	1.66 × 10^−10^	1.05–1.09
NO_2_–2010	13,957	49,184	26.73 (7.70)	1.081	3.43 × 10^−13^	1.06–1.10
NO_2_–2007	13,957	49,184	31.53 (11.24)	1.035	0.001	1.01–1.06
NO_2_–2006	13,957	49,184	29.43 (9.60)	1.068	5.23 × 10^−10^	1.04–1.09
NO_2_–2005	13,957	49,184	30.56 (10.60)	1.048	8.95 × 10^−6^	1.02–1.07

Covariates: Age, Sex, 5 principal components and Index of Multiple Deprivation. Units for all variables: micrograms/m^3^.

**Table 4:** Effect of Green Space.

Measure	Cases	Controls	Covariates	Mean (SD)	Odds Ratio	*p*-Value	95% CI
Green Space 300 m	13,941	49,330	IMD	35.38 (23.12)	0.925	1.99 × 10^−13^	0.906–0.944
Green Space 300 m	14,223	50,111	PM2.5, NOx	35.33 (23.04)	0.961	0.002	0.937–0.985
Green Space 1000 m	13,941	49,330	IMD	45.14 (21.58)	0.940	7.85 × 10^−9^	0.920–0.960
Green Space 1000 m	14,223	50,111	PM2.5, NOx	45.08 (21.53)	0.957	6.29 × 10^−4^	0.933–0.981
Natural Environment 300 m	13,941	49,331	IMD	26.44 (25.37)	0.939	3.57 × 10^−9^	0.920–0.960
Natural Environment 300 m	14,836	50,503	PM2.5, NOx	26.37 (25.25)	0.974	0.04	0.950–0.999
Natural Environment 1000 m	13,941	49,331	IMD	40.92 (25.82)	0.941	2.70 × 10^−8^	0.921–0.961
Natural Environment 1000 m	14,836	50,503	PM2.5, NOx	40.86 (25.76)	0.952	1.76 × 10^−4^	0.928–0.977

Covariates: Age, Sex, 5 principal components for all models in addition to those listed. Unit of measurement is in percent.

## Data Availability

All data analyzed in this research is from the UK Biobank Resource and is available through the UK Biobank’s application process (https://www.ukbiobank.ac.uk/enable-your-research/apply-for-access; accessed on 25 March 2021).
